# Roman Catholic Inebriates and Their Religion

**Published:** 1903-10-31

**Authors:** 


### Editor's Letter-Box.

**[Our Correspondents are reminded that prolixity is a great bar to publication, and that brevity of style and conciseness of statement greatly facilitate early insertion.]**

### ROMAN CATHOLIC INEBRIATES AND THEIR RELIGION.

The Hon. Secretary of the Royal Victoria Homes, Brentry, writes: Thank you for calling my attention to the remarks in your issue of the 10th inst. The difficulty at Brentry has now, I am glad to say, been removed.

Up to the first week in December, 1902, the services of a part time Roman Catholic priest were available, but at that date they were withdrawn, the Roman Catholic authorities refusing to continue them unless the Board increased the stipend of the priest. The Board were paying the priest at the same rate as part time priests are paid in all Government establishments, and in addition to the stipend the Board paid the priest's cab expenses between the convent (situated 1½ miles distance) and the homes, and seeing no reason why more should be paid at Brentry than elsewhere, the Board declined to increase the stipend. A considerable amount of correspondence followed, which has now ended, and the Roman Catholic authorities have recommenced their work on the same stipend as heretofore. The only difference in the terms being the priest is to be paid an additional sum of £10 per annum and to provide his own cabs or other means of conveyance instead of the Board paying for them.

